# Books and Authors

**Published:** 1907-01

**Authors:** 


### BOOKS AND AUTHORS.

**Surgery.** Its Principles and Practice. By various authors. Edited by **William Williams Keen,** M.D., Professor of the Principles of Surgery and of Clinical Surgery in Jefferson Medical College, Philadelphia. In 5 volumes. Volume I. History, Surgical Physiology, Surgical Pathology, Infections, Tumors, Wounds. Large Octavo, pp. 983. With 261 text illustrations and 17 colored plates. Philadelphia and London: W. B. Saunders Co. 1906. (Price, cloth, $7.00; half morocco, $8.00, net prices).

The attractiveness of the field of surgery furnishes an inducement to both authors and publishers to prepare and print gigantic works, encyclopedic in character and exhaustive in method. The scheme of this treatise is to present a series of monographs, each complete in itself, by surgeons in various parts of the world, more or less distinguished in the subjects delineated, and which when completed shall be a practical exposition of modern surgical treatment.

This volume consists of twenty-two chapters and an index. Each chapter deals with a fundamental topic, such as, for example, inflammation, examination of the blood, gangrene, the process of repair, tuberculosis, and the like, and is complete in itself. The contributors are, J. George Adami, John Bland-Sutton, George W. Crile, John Chalmers Da Costa, John C. Da Costa, Jr., Charles Harrison Frazier, Leonard Freeman, Ludvig Hektoen, Edward Martin, James Gregory Mumford, Edward Hall Nichols, Eugene Alfred Smith, and Francis Carter Wood.

The chapter on traumatic fevers is written by Eugene A. Smith, of Buffalo, and is one of the features of the book. Dr. Smith attacks the faulty nomenclature applied to these fevers and proposes a simplified classification. He would do away with many terms which are used indiscriminately, such, for example, as sapremia, blood-poisoning, autoinfection, septicemia, and the like, as incorrect, misleading, and unnecessary. He gives good and sufficient reasons for discarding these terms, and offers in their stead as a simpler classification the division of traumatic fevers into two general varieties: the aseptic, and the septic. Theoretically and clinically, he says, the septic type admits of ready subdivision into two forms,—septicemia and pyemia. He would not coin new terms but he would limit and define the terms we now employ.

Dr. Smith has written a scholarly disquisition on this topic which every surgeon,—even every physician,—should read with care. It puts a new color on an old subject, freshens and rejuvenates it, and makes it read like a story; and, withal, it is so fascinating that one cannot put it aside until he has finished reading it in its entirety.

The chapter on surgical tuberculosis by the senior Da Costa is another monograph of special interest. Rarely has so much scientific material relating to this topic been presented in such condensed form, all the later views being set forth, and the history of surgical tuberculosis, also, being related from our earliest knowledge of the subject. The final chapter, which deals with wounds and contusions is written by George W. Crile, and is still another section of the work that deserves special mention. It is quite unnecessary to go into detail as to the merits of this chapter but it deserves thoughtful reading as a whole, while the subtopic, shock and collapse, is worthy of study and deserving of praise.

The editor is a surgeon of such large experience, a teacher of such renown, and an author of such fame that it could not be otherwise than that he should put forth a superior treatise. The work is carefully edited, the illustrations are artistic, and the printing is excellent. When completed, it is destined to take permanent place in surgical literature.

---

**The Prophylaxis and Treatment of Internal Diseases.** By **F. Forchheimer,** M.D., Professor of Theory and Practice of Medicine and Clinical Medicine in the Medical College of Ohio (University of Cincinnati). Octavo, pp. 669. New York and London: D. Appleton & Co. 1906. (Price, $5.00, net).

The prevention of disease is the special prerogative of the twentieth century physician. It is, therefore, of the utmost importance that he should make himself familiar with every phase of prophylaxis, in order that he may apply its principles to all conditions at a moment's notice.

The various diseases are dealt with in their natural groups. First, come the infectious diseases; second, the diseases produced by animal parasites; third, the intoxications; fourth, constitutional diseases; fifth, diseases of the digestive system; sixth, diseases of the respiratory apparatus; seventh, diseases of the circulatory system; eighth, diseases of the blood and the ductless glands; ninth, diseases of the kidneys; tenth, diseases of the bladder; eleventh, diseases of the male sexual organs; twelfth, diseases of the nervous system.

Each individual disease in its respective group is taken up and its prophylaxis and treatment are set forth succinctly, sometimes tersely, but always intelligently. Especially in the line of treatment are the methods laid down those of later experiences. Care has been taken, also, to recommend whenever possible those methods best adapted to private practice. It is in all its details a work that is suited to the requirements of an active daily professional life,—to the physician we have become accustomed to hear somewhat tritely called a "busy doctor."

It is, moreover, a work of great breadth and is written by a man of extensive clinical experience, a resourceful teacher of enlarged views, capable of imparting his knowledge to others without unnecessary verbiage, but with a forcefulness that makes it lasting and a clearness that lends a charm to its reading or study.

Its claims may be thus summarised: it is in a class by itself, it being the first book of its kind published in this country; it is both a practical and a scientific exposition of the topics with which it deals; it is a safe guide and may be accepted as such with perfect confidence; it is a complete digest of the most modern methods of preventive and curative medicine; and, finally, it fills the gap in literature between the manual of treatment and the textbook of practice. It is complete in itself and will be in demand by the general practitioner of medicine who desires to keep pace with the latest thought concerning the prevention and treatment of disease.

---

**Medical Jurisprudence, Forsenic Medicine, and Toxicology.** By **R. A. Witthaus**, A.M., M.D., Professor of Chemistry, Physics and Toxicology in Cornell University; and **Tracy C. Becker**, A.B., LL.B., Professor of Criminal Law and Medical Jurisprudence in the University of Buffalo. Vol. I. Octavo, pp. LV-996. Second edition, New York: William Wood & Co. 1906. (Muslin, $6.00; Law sheep, $7.00, net prices).

It is twelve years since the first edition of this great work was issued and though changes in this department of medical science are not as speedy as in most others, yet they have been sufficient to justify some revisions, some additions, and some eliminations. The general style and makeup of the work, however, remain unchanged and this volume might easily be mistaken for the first volume of the former edition.

The first section of the text in this book is written by Mr. Becker and deals with the legal relations of physicians and surgeons. This topic includes the acquirement of the right to practise medicine, their legal duties and obligations, their right to compensation, their privileges and duties when summoned as witnesses in courts of justice, and their liability for malpractice. Every physician should familiarise himself with the six chapters of this section. The next section, written by Mr. Boston, of the New York bar, presents the law of evidence concerning communications between patient and physician. Mr. Boston also has prepared a synopsis of the laws regulating the practice of medicine in the United States, Great Britain and British North America, from the latest statutes, with annotations. This synopsis constitutes a section of about 600 pages, and is the most complete compilation yet published. It is an invaluable record for reference.

The next section is written by Mr. Becker and relates to the legal status of the dead body. It comprises questions regarding the disposal and obligations to dispose of the body, how and by whom it may be exhumed or removed; autopsies and by whom they may be ordered; and the rights of relatives and accused persons. An appendix to this section contains a synopsis of the statutes of the different states and territories concerning these several questions. A section follows on medico-legal autopsies, by H. P. Loomis; and then comes a section on personal identity, by Irving C. Rosse; another, by H. P. Loomis, on the determination of the time of death; and, finally, two sections by E. V. Stoddard entitled respectively, death by heat and cold, and death by starvation.

The foot notes are very elaborate and voluminous adding very much to the value of the treatise and facilitating reference to other works. It is an indispensable treatise for lawyer, physician and chemist, and speaks with authority on the topics of medical jurisprudence, forensic medicine, and toxicology.

---

**International Clinics.** A quarterly of Illustrated Clinical Lectures and especially prepared articles on Treatment, Medicine, Surgery, Neurology, Pediatrics, Obstetrics, Gynecology, Orthopedics, Pathology, Dermatology, Ophthalmology, Otology, Rhinology, Laryngology, Hygiene and other topics of interest to students and practitioners. By leading members of the medical profession throughout the world. Edited by **A. O. J. Kelly**, A.M., M.D. Volume III. Sixteenth series. 1906. Philadelphia and London: J. B. Lippincott Co. (Cloth, $2.00).

This number is rich in quality and quantity. Four articles on treatment are distributed as follows: treatment of acute pleurisy, A. A. Stevens; treatment of certain forms of bronchitis, Joseph M. Patton; treatment of dilatation of the heart, William H.

Katzenbach; Fournier's recent modification of his treatment of syphilis, H. Saingery.

The articles on medicine are thus distributed: autointoxications and their treatment, Edward C. Hill; multiple ulcers of the pylorus and gastrosuccorrhea (Reichmann's disease) without stagnation in the stomach, Maurice Soupault; accidental rashes occurring in the course of 250 consecutive cases of typhoid fever, Dr. J. Milton Miller; the irregular heart—its causes and treatment, Bertram Abrahams; diagnosis of some chronic joint affections, H. S. Clogg; predisposition to, and prevention and treatment of, pulmonary tuberculosis, John W. Wainwright; milky urine, F. X. Gouraud; life in the antarctic from a medical point of view, J. H. Harvey Pirie.

In the department of surgery we find the following articles: hyperemia treatment of swollen joints, E. H. Bradford; surgical complications of pneumonia and their treatment, T. Turner Thomas; preliminary note on a rational operation for the radical cure of inguinal hernia, A. N. McGregor; inguinal hernia in the female, E. Scott Carmichael; clinical significance of peritoneal adhesions, Charles Greene Cumston; hemorrhagic diathesis complicating surgical work, Frederick Griffith; surgical results which may follow improper feeding, Edred M. Corner; disorders of the umbilicus with special reference to the newborn and infant.—II. Umbilical fistulas, sinuses, and cysts, A. Ernest Gallant.

In obstetrics and gynecology the articles are: tubal pregnancy terminating in spontaneous recovery without operation, (also several other titles), Thomas J. Watkins; use of forceps in deep transverse arrest of the head, Charles B. Reed; the pelvis of lame women, A. Pinard. In rhinology there is one article entitled, clinical rhinometry—functional examination of the nose, Marcel Lermoyez; in otology one article entitled, primary thrombosis of the jugular bulb, Barton H. Potts, and in pathology one article entitled, leukemia and sarcomatosis, G. Banti. This exhibit of the contents and the authors will convey an intelligent idea of the value of the book. It is exceptionally well illustrated and becomes an important part of the medical literature of the year.

---

**The Practice of Pediatrics by American and English Authors.** Edited by **Walter Lester Carr,** M.D., Consulting Physician to the French Hospital; Visiting Physician to the Infants' and Children's Hospital, New York. In one octavo volume of 1014 pages with 199 engravings and 32 full page plates in colors and monochrome. Philadelphia and New York: Lea Brothers & Co. 1906. (Cloth, $6.00; leather, $7.00; half morocco, $8.00, net prices).

This is the second number issued of the series known as the practitioners' library, embracing the fields of gynecology, obstetrics, and pediatrics. It is, we believe, the first time this special topic has been dealt with in encyclopedic form. The contributors for the most part, are distinguished pediatrists in

America and England and their opinions are entitled to that respect which experience and knowledge engender.

The work is essentially clinical in character and is distinguished for the emphasis given to treatment. Infant feeding, a knowledge of which is so important in the intelligent care of children sick or well, is given prominence, and diseases of the alimentary tract are presented in much detail. Disorders of nutrition, so frequently obscure and difficult to manage, are presented with special clearness; diseases of the circulation and of respiration are handled with great skill; contagious diseases, so important as to early diagnosis, receive that careful description which is so needful to their recognition and management; diseases of the nervous system, of the glands, of the lymphatic system, and of the skin, are all set forth in the most modern fashion.

Diagnosis is given that prominence all through the book which its great importance requires and the employment of the best methods is insisted upon. Physicians skilled in internal medicine, in therapeutics, in obstetrics, and in dermatology have been laid under contribution, the result being a most comprehensive and thoroughly modern treatise,—one that will take first place as a book of reference for the practitioner as well as a guide to the clinician. The illustrations are of a character that give increased value to the text.

---

**Modern Clinical Medicine. Vol. III.** Diseases of the Digestive System. Edited by Frank Billings, M.D., Professor of Medicine, University of Chicago; Professor of Medicine and Dean of Faculty, Rush Medical College. Octavo, pp. xvi-824. With 45 illustrations. New York and London: D. Appleton & Co. 1906.

It is a fortunate circumstance that Professor Billings was prevailed upon to edit this volume, for certainly no American physician is more competent to deal with diseases of the digestive system. It is the third volume in the series of modern clinical medicine, and should receive the attention of every clinician who teaches internal medicine.

There is less uncertainty nowadays in the diagnosis and treatment of these affections since much that formerly was theoretical has passed into the realm of established fact. Chemistry has done much to accomplish this result, while the study of metabolism has contributed greatly to clear up doubt. The functions and diseases of the pancreas are under the searchlight of investigation, while the study of the feces is being pursued to an extent never thought possible or important, but with marvelous results in relation to diseases of the intestinal tract.

These studies and investigations have resulted in making such a volume as this possible, and its benefits to mankind will be far-reaching as well as lasting. It must not be forgotten, moreover, that abdominal surgery has thrown much light on the problems dealt with in this book and has led to the solution of some of the most difficult of them. It is a twentieth century work in every sense of the word and cannot fail to take its place in the forefront of the best medical literature of the day.

---

**A Treatise on the Motor Apparatus of the Eyes.** Embracing an Exposition of the Anomalies of the Ocular Adjustments and their Treatment, with the Anatomy and Physiology of the Muscles and their Accessories. By George T. Stevens, M.D., Ph.D. Illustrated with 184 engravings, some in colors. 496 pages, Royal Octavo. Philadelphia: F. A. Davis Company. 1906. (Price, $4.50 net).

In these days of work and worry, eyestrain plays no inconsiderable part as a cause of the nerve tire that results from the strenuous life of the period. The author of this book is an ophthalmologist of great experience and has presented a work that will command the attention of everyone who gives special study to the eye or who makes its diseases a special line of practice.

The book is replete with ingenious tests to determine anomalies of the motor apparatus and of equally ingenious devices for their correction. Dr. Stevens enters into minute detail in the descriptions of his methods and of the instruments which he has devised or modified for their application. Much that here appears he has presented heretofore in the form of monographs or society papers; much, also, is published for the first time, all of which is worked into a homogenious, compact form, that becomes an interesting and instructive exposition of the author's views and methods of practice.

It is a handsome book, beautifully illustrated, and a valuable addition to the literature of ophthalmology.

---

**The Practitioners' Visiting List for 1907.** A pocket-sized book containing memoranda and data important for every physician, and ruled blanks for recording the details of practice. The weekly, monthly and 30-patient perpetual contain 32 pages of data and 160 pages of classified blanks. The 60-patient perpetual consists of 256 pages of blanks alone. Each in one wallet-shaped book, bound in flexible leather, with flap and pocket, pencil and rubber, and calendar for two years. Price by mail, postpaid, to any address, $1.25. Thumb-letter index, 25 cents extra. Philadelphia and New York: Lea Brothers & Co., Publishers. 1906.

This excellent book, the successor of the Medical News visiting list, is so accurately described in the heading prepared by the publishers, that very little remains to be offered by the writer. It is proper to remark, however, that it is in its twenty-first year of publication, hence contains all that such a long experience indicates as essential for the daily needs of every busy physician. The text has been revised to meet present conditions and contains, among other things, a scheme of dentition; tables of weights and measures and comparative scales; instructions for examining the urine; diagnostic table of eruptive fevers; incompatibles, poisons and antidotes; directions for effecting artificial respiration; extensive table of doses; an alphabetical table of diseases and their remedies, and directions for ligation of arteries.

The record portion contains ruled blanks of various kinds, adapted for noting all details of practice and professional business. It is difficult to see how a book of this kind could be more complete.

---

**The Practical Medicine Series of Year Books.** Ten Volumes. Issued under the general editorial charge of **Gustavus P. Head,** M.D., Professor of Laryngology and Rhinology in the Chicago Post-Graduate Medical School. Vols. IV and V., series of 1906. Chicago: The Year Book Publishers. 1906. (Prices, $1.50, $1.25; entire series, $10.00). Vol. IV. Gynecology. Edited by **Emilius C. Dudley,** A.M., M.D., and **C. von Bachellé,** M.S., M.D.

This interesting resume of the additions of value to the literature of gynecology for the year 1905, is prepared by men of experience who are accustomed to sort the vast material offered with careful judgment. Early diagnosis in uterine carcinoma receives marked attention, while Dudley's management of urinary incontinence is described and illustrated. Various other operations are presented, such as perineorrhaphy, displacements, tumors of the vulva, and the like, all of which are admirably illustrated. It is the best gynecological abstract we have met, and should be prized by all who practice in this field.

**Vol. V, Obstetrics.** Edited by **Joseph B. De Lee,** A.M., M.D., with the collaboration of **D. Roehler,** M.D., and **Herbert M. Stowe,** M.D.

In this book vaginal Cesarean section is admirably delineated, both in text and illustration; the complications of pregnancy are worked out to considerable extent; eclampsia is still undergoing investigation; while operative obstetrics is given due prominence. The puerperal infections receive considerable attention, especially their surgical management, and the diseases of the newborn are appropriately dealt with. Every obstetrician will derive benefit from this grouping of obstetrical progress, which has been done with the intelligent brain and hand of a master, the practical side of the subject being kept constantly in view.

---

**Thornton's Pocket Medical Formulary** (heretofore known as The Medical News Pocket Formulary). New (7th) edition, revised to accord with the new U. S. Pharmacopeia, containing more than 2,000 prescriptions with indications for their use. In one leather bound volume. Philadelphia and New York: Lea, Brothers & Co., Publishers. 1906. (Price, $1.50 net).

This useful reminder contains many valuable hints, and may well be kept at hand by every busy physician. The changes in the new pharmacopeia have been conformed to, and other revisions have been made, making it in every way a present-day prescription book. It also contains a table of doses, a list of incompatibles, a summary of poisons and antidotes, as well as much other information that may be drawn upon to refresh the memory in the presence of an emergency. It is in reality a scientifically arranged book of formulæ, with annotations giving indications for the use of each prescription, and is full of valuable suggestions.

**Lea's Series of Medical Epitomes.** Edited by **Victor C. Pedersen,** M.D. Materia Medica and Therapeutics by **Edward J. Kiepe,** M.D., Adjunct Professor of Materia Medica in the Medical Department, University of Buffalo. 12mo., pp. 265. Philadelphia and New York: Lea Brothers & Co. 1906. (Price $1.00).

The topic dealt with in this book is one that can be presented in epitome form with considerable clearness. Professor Kiepe certainly has made a very attractive little manual, giving the essentials of his subject in direct fashion, without superfluous verbiage. The directions relating to prescription writing are excellent and may be studied by some of the older members of the profession with propriety. The drugs described are arranged in alphabetical order, which makes them easily accessible. The eighth decennial revision of the pharmacopeia has been conformed to as far as applicable, and the entire work shows a finish that is praiseworthy.

---

**Blakiston's Quiz Compends.** Genitourinary Diseases and Syphilis. By **Charles S. Hirsch,** M.D., Assistant in the Genitourinary surgical department, Jefferson Medical College. 12mo., pp. 351. Illustrated. Philadelphia: P. Blakiston's Son & Co. 1906. (Price, $1.00).

Hirsch has made an excellent condensation of these subjects and the book will be welcomed both by students and quiz masters. The most that can be expected to be accomplished by such a book is to gain an abstract of the essentials, leaving the minute details to the textbooks. A vast amount of useful material, however, has been grouped in these pages which will serve to lighten the task of those for whom the book is intended. The practitioner, too, can even find hints in it that will not come amiss during his busy work.

---

**Annual report of the Department of Health of the City of Buffalo;** for the year ending December 31, 1905. Walter D. Greene, M.D., Health Commissioner. Buffalo: Hausauer-Jones Printing Company. 1906.

The annual death rate during 1905, according to this report, was 14.14 per 1,000. The tables show a decrease in communicable diseases, and, contrary to the general belief, a lower death rate from tuberculosis than that of most large cities. The mortality rate of the first five years of life is indicated to be 4.05,—a remarkable showing. These and various other facts exhibited by this report testify to the efficiency of the department in a conclusive manner. The time is ripe, however, for the health department to consider the propriety of making war on the smoke and noise nuisances, and it should take action to prevent spitting on the sidewalks. A crusade against these three intolerable conditions would be a credit to the city.

